# Cerebellar transcranial alternating current stimulation in the gamma range applied during the acquisition of a novel motor skill

**DOI:** 10.1038/s41598-020-68028-9

**Published:** 2020-07-08

**Authors:** Maximilian J. Wessel, Laurijn R. Draaisma, Anne F. W. de Boer, Chang-hyun Park, Pablo Maceira-Elvira, Manon Durand-Ruel, Philipp J. Koch, Takuya Morishita, Friedhelm C. Hummel

**Affiliations:** 1 Defitech Chair of Clinical Neuroengineering, Center for Neuroprosthetics (CNP) and Brain Mind Institute (BMI), Swiss Federal Institute of Technology (EPFL), Campus Biotech, Chemin des Mines 9, 1202 Geneva, Switzerland; 2 Defitech Chair of Clinical Neuroengineering, Clinique Romande de Réadaptation, Center for Neuroprosthetics (CNP) and Brain Mind Institute (BMI), Swiss Federal Institute of Technology (EPFL Valais), Sion, Switzerland; 3 Clinical Neuroscience, University of Geneva Medical School, Geneva, Switzerland

**Keywords:** Learning and memory, Neurology

## Abstract

The development of novel strategies to augment motor training success is of great interest for healthy persons and neurological patients. A promising approach is the combination of training with transcranial electric stimulation. However, limited reproducibility and varying effect sizes make further protocol optimization necessary. We tested the effects of a novel cerebellar transcranial alternating current stimulation protocol (tACS) on motor skill learning. Furthermore, we studied underlying mechanisms by means of transcranial magnetic stimulation and analysis of fMRI-based resting-state connectivity. N = 15 young, healthy participants were recruited. 50 Hz tACS was applied to the left cerebellum in a double-blind, sham-controlled, cross-over design concurrently to the acquisition of a novel motor skill. Potential underlying mechanisms were assessed by studying short intracortical inhibition at rest (SICI_rest_) and in the premovement phase (SICI_move_), intracortical facilitation at rest (ICF_rest_), and seed-based resting-state fMRI-based functional connectivity (FC) in a hypothesis-driven motor learning network. Active stimulation did not enhance skill acquisition or retention. Minor effects on striato-parietal FC were present. Linear mixed effects modelling identified SICI_move_ modulation and baseline task performance as the most influential determining factors for predicting training success. Accounting for the identified factors may allow to stratify participants for future training-based interventions.

## Introduction

Neurostimulation in combination with behavioural training is a promising strategy for restoring normal function after neuronal damage or for functional enhancement in healthy individuals^1^. Currently, numerous interventional studies are implementing this strategy by combining transcranial electric stimulation (tES) with motor training. First evidence for this approach provided promising results, for review please see e.g., work from Wessel and colleagues or Buch and colleagues^2,3^. Proof-of-principle studies could show that the application of transcranial direct current stimulation (tDCS) to the primary motor cortex (M1) could enhance the learning of a novel motor skill in healthy individuals or chronic stroke patients^4,5^. However, several challenges, which limit further translation of this approach remain, such as limited reproducibility, varying and non-satisfactory effect sizes, and a lack of mechanistic understanding^3^.

To partially address these challenges, we strived to validate potential effects of a novel cerebellar transcranial alternating current (tACS) protocol^6^ on the acquisition of a novel motor skill in healthy individuals. We based our research approach on three main assumptions. Firstly, the cerebellum is considered as a core node of the motor learning network^7,8^ and it has a high potential to undergo neuroplastic changes^9,10^. These plastic changes can be modulated by tES techniques^11–13^. Secondly, tACS, when compared with steady-state stimulation protocols such as tDCS, offers the benefit of exerting its effects more selectively on targeted neuronal elements, which have an “Eigenfrequency” close to the stimulation frequency^14,15^. Furthermore, oscillatory activity in cerebellar cortex has been linked to various aspects of neuronal processing^16^. We specifically chose a 50 Hz stimulation frequency, based on the work from Naro and colleagues, who have demonstrated facilitatory effects on different aspects of motor function^6,15^, namely repetitive finger opposition movements or some items of the Wolf Motor Function Test (WMFT) such as turning a key in a lock. It is of note however, that the field of cerebellar tACS is just evolving and more research and validation also studying other stimulation frequencies is needed. Thirdly, we investigated potential underlying mechanisms by studying intracortical interactions in M1 with double-pulse transcranial magnetic stimulation (dpTMS) to determine intracortical inhibitory and facilitatory neurotransmission. Specifically, we chose to investigate short intracortical inhibition (SICI) and intracortical facilitation (ICF) allowing us to evaluate markers of GABAergic and glutamatergic neurotransmission^17^, which have been linked to memory formation and motor learning^18–20^. Furthermore, SICI and ICF interact with the cerebello-cortical output drive^21^, which has shown to be responsive to modulation via 50 Hz tACS^6^. We assessed seed-based functional connectivity (FC)^22^ in a hypothesis driven motor learning network^8^ with resting-state functional magnetic resonance imaging (rsfMRI), allowing us to extend our research perspective towards also studying effects on larger scale brain network interactions. We strived to combine this multi-modal data to create predictive models for the responsiveness towards our intervention or training, which may allow us to stratify participants in responders and non-responders in future studies and hereby reduce variability and stabilize effect sizes.

In the present study, we hypothesized that: (i) active cerebellar tACS applied concurrently to the training phase enhances the acquisition of the novel motor skill, and (ii) a combined predictive model including multi-modal parameters from behavioural motor tests, dpTMS, and rsfMRI assessments will allow to predict the learning success.

## Results

### tACS-associated sensations and effectiveness of blinding

All participants tolerated the tACS application well, there were no adverse effects. Minor stimulation-associated sensations were present, see Supplementary Table S1. The participants’ ability to distinguish active from sham stimulation was not significantly different from chance level. Please see also Supplementary Information and Supplementary Table S1.

### Behavioural data

**Figure 1:**
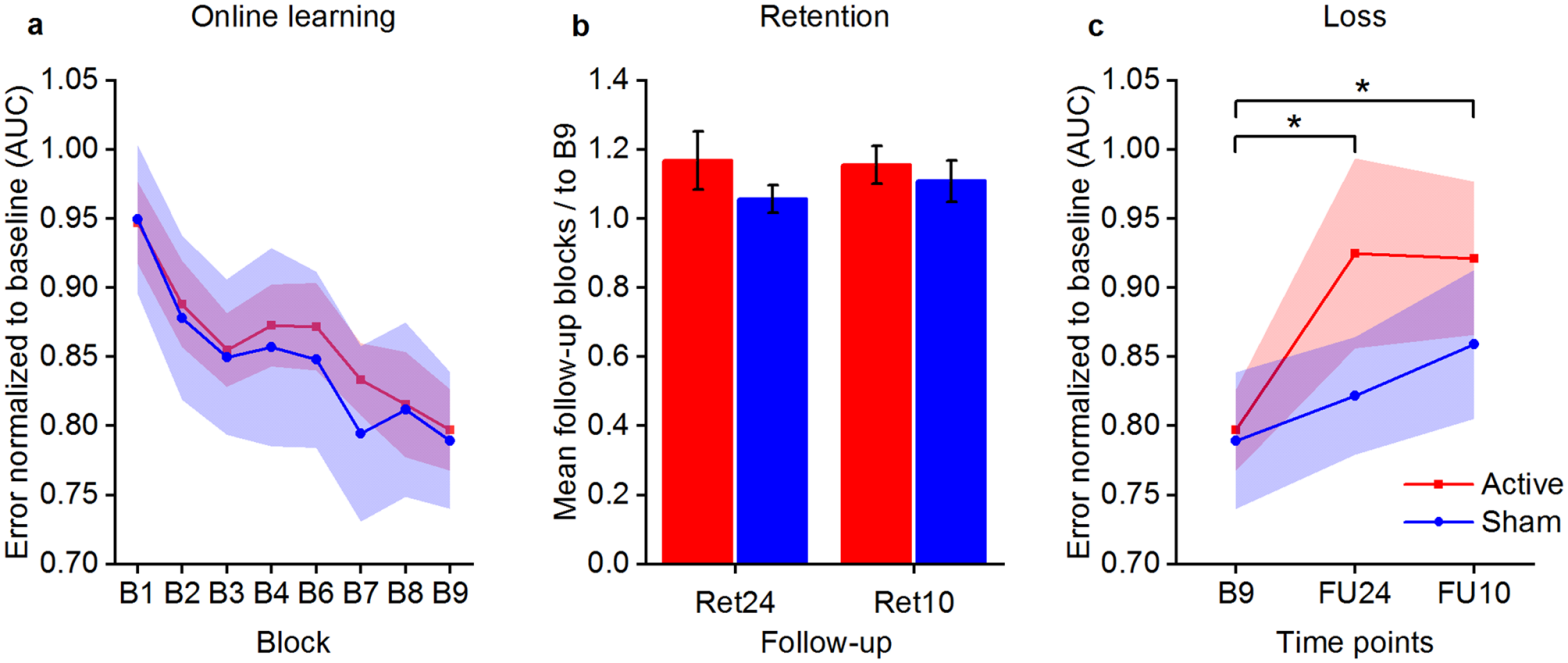
Behavioural data. (**a**) Training performance during the motor task normalized to baseline, quantified as area under the curve (AUC) of the movement trajectory of correctly performed sequences. Block B5 (not shown) was used as a performance probe, applying a pseudorandom, untrained sequence. (**b**) Retention measured circa 24 h (Ret24) and circa 10 days (Ret10) after training. Retention was calculation by contrasting the average the follow-up blocks to the last training block (B9). (**c**) Figure depicts performance during the two follow-up sessions (FU24 and FU10) compared and the last block of training (B9). Follow-up performance was averaged per session. Margin of errors are depicted as standard error of the mean (s.e.m.).

The analysis of the training phase revealed a significant effect of BLOCK χ(7) = 36.65, *p* < 0.001, f^2^ = 0.071, but not of STIMULATION χ(1) = 3.14, *p* = 0.076, f^2^ = 0.008, or BLOCK x STIMULATION interaction χ(7) = 0.76, *p* = 0.998, f^2^ = 0.001, indicating that our learning task—the sequential grip force modulation task (SGFMT)—served as a valid learning model. The potential null result for STIMULATION suggested by the frequentist analysis was further examined by additionally conducting a Bayesian-based analysis. The Bayesian repeated measures ANOVA indicated a Bayes factor for STIMULATION of BF_01_ = 5.85, meaning that the data was 5.85 times more likely to occur under the null-hypothesis than the alternative hypothesis. This is considered a moderate effect. The Bayes factor for BLOCK was BF_01_ = 1.95, which corresponds to a small effect. Additionally, the Bayes factor for the interaction between BLOCK and STIMULATION was BF_01_ = 649.59, meaning that it was 649.59 times more likely that the null-hypothesis was true. This is considered a strong effect. The combined statistical approaches indicate that no clear stimulation associated effects were present during the training phase, see Fig. 1a. The pseudorandom block (B5) did not significantly differ from the neighbouring blocks, B4 vs. B5 t(23) = 1.28, *p* = 0.214, *d* = 0.26, B5 vs. B6 t(23) = 1.10, *p* = 0.283, *d* = 0.22, implying that also sequence-independent learning was present.

In order to avoid carry-over effects there was a wash-out period between the before and after cross-over sessions of M: 50.8 (SD: 32.43) days with a range between 25 and 103 days. The comparison of baseline performance before and after cross-over did not indicate major carry-over effects t(21.28) = 0.22, *p* = 0.826, *d* = 0.09.

Retention at T3 and T4 were analysed as compound measures (RETENTION) using the average of the three blocks per session divided by the last training block (B9). The analysis of the retention phase indicated a significant effect for STIMULATION χ(1) = 6.50, *p* = 0.011, f^2^ = 0.084, but not of RETENTION χ(1) = 0.26, *p* = 0.609, f^2^ = 0.003, or RETENTION x STIMULATION interaction χ(1) = 0.71, *p* = 0.399, f^2^ = 0.007 for the whole group analysis, see Fig. 1b. However, the significant STIMULATION effect did not persist after performing an influential point analysis (leave-one-out approach)—STIMULATION χ(1) = 2.72, *p* = 0.099, f^2^ = 0.044. With the same reasoning as for the training data, we performed a Bayesian ANOVA on the retention data to investigate the potential null result in more depth. The result showed a Bayes factor for STIMULATION of BF_01_ = 1.73, meaning that it was 1.73 times more likely that the null-hypothesis (no stimulation effect) was true. This is considered a small effect, which indicates that the analysis does not provide substantial evidence for the null-hypothesis (no effect of stimulation on retention) considering the commonly accepted threshold of BF_01_ > 3^23^. However, under the assumption of minor, slight stimulation-associated effects on retention these were rather in the direction of task disturbance. Moreover, the Bayes factor for RETENTION was BF_01_ = 3.34, showing that it was 3.34 times more likely that the null-hypothesis (no effect of time) was true, this is considered a moderate effect. The interaction between STIMULATION x RETENTION resulted in a Bayes factor of BF_01_ = 14.83, rendering it 14.83 times more likely that the null-hypothesis was true over the alternative hypothesis. In conclusion, we observed a slight indication for disturbance of skill retention by 50 Hz cerebellar tACS, which however could not be confirmed after correcting for an influential data point.

Next, we proceeded by analysing the magnitude of the skill reduction at the follow-up assessments by contrasting their session average to the last training block (B9). Active and sham groups showed a significant loss when compared with B9 χ(2) = 6.38, *p* = 0.041, f^2^ = 0.058. There was no significant effect of STIMULATION χ(1) = 1.23, *p* = 0.289, f^2^ = 0.013 or SESSION x STIMULATION interaction χ(2) = 1.46, *p* = 0.481, f^2^ = 0.012, see Fig. 1c.

### Short intracortical inhibition (SICI_rest_) and intracortical facilitation (ICF_rest_) at rest

**Figure 2:**
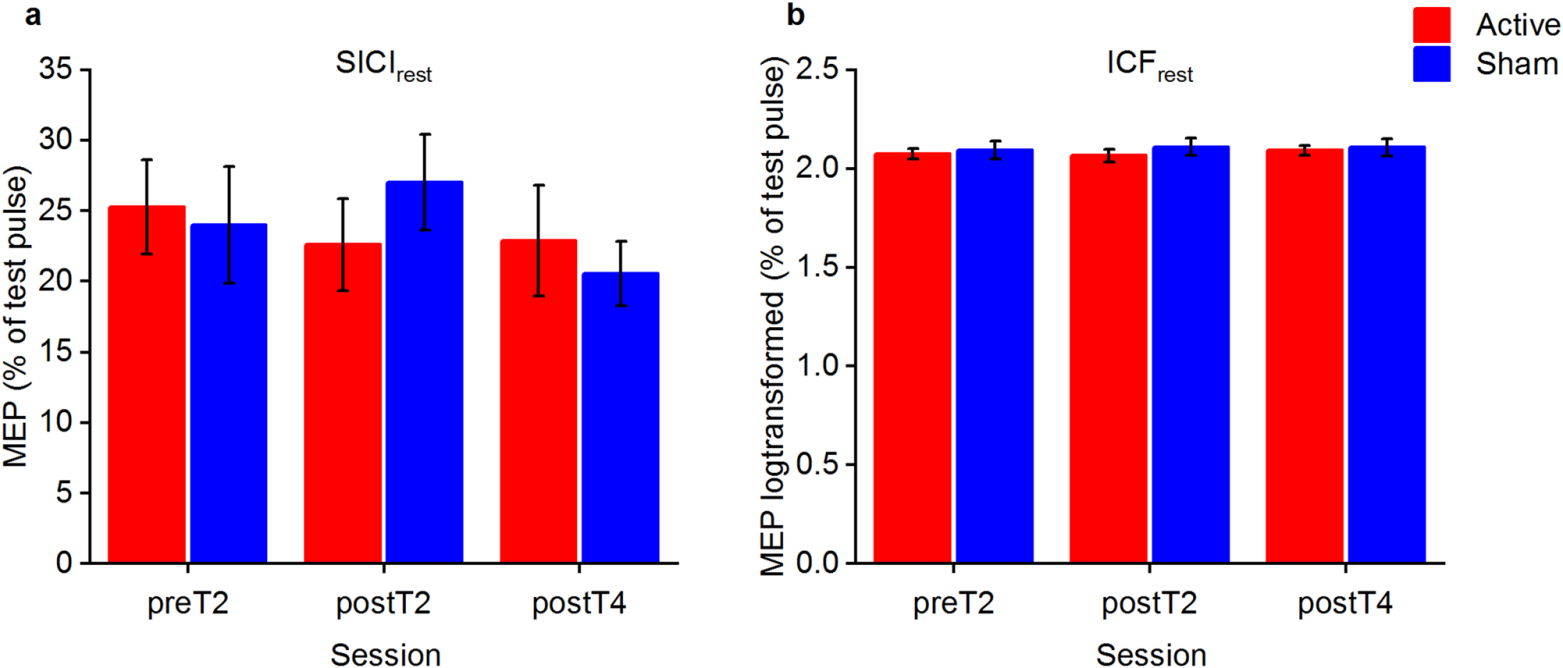
SICI_rest_ and ICF_rest_. (**a**) SICI measured with dpTMS during rest. MEP is related to average test pulse. (**b**) ICF measured during rest. Data was log-transformed to achieve homogeneity of variance. MEP is related to average test pulse. Error bars are depicted as standard error of the mean (s.e.m.).

Test pulse (TP) peak-to-peak amplitudes at rest and required percentage of maximal stimulator output (MSO) were not significantly different between stimulation conditions or assessment time points, confirming a stable TP adjustment throughout the course of the experiment, for details please see Table 1. The analysis of the SICI_rest_ data revealed no effect of STIMULATION χ(1) = 0.01, *p* = 0.916, f^2^ < 0.001, SESSION χ(2) = 2.05, *p* = 0.359, f^2^ = 0.013 or STIMULATION x SESSION interaction χ(2) = 2.30, *p* = 0.316, f^2^ = 0.014, see Fig. 2a. To achieve homogeneity of variance the ICF_rest_ data were log-transformed. The analysis of ICF_rest_ indicated no effect of STIMULATION χ(1) = 1.30, *p* = 0.254, f^2^ = 0.007, SESSION χ(2) = 0.31, *p* = 0.857, f^2^ = 0.002 or STIMULATION x SESSION interaction χ(2) = 0.62, *p* = 0.732, f^2^ = 0.003, see Fig. 2b. In summary, our dataset failed to reveal any significant differences in the modulation of intracortical interactions in M1 at rest by phase of learning or stimulation type.

**Table 1:** Averages of peak-to-peak amplitudes of single pulse MEP’s at resting state, measured in millivolts (mV) and the percentage (%) of maximal stimulator output (MSO) that was used to induce the single pulse MEPs, s.e.m. are depicted between brackets.

Stimulation	PreT2	PostT2	PostT4	Statistics
**Peak-to-peak amplitudes of TP**_**only**_** MEPs (mV)**				
Active	1.44 (0.16)	1.46 (0.17)	1.46 (0.15)	STIMULATION χ(1) = 0.40, * p* = 0.529; f^2^ = 0.002; SESSION χ(2) = 2.71, * p* = 0.258, f^2^ = 0.015; STIMULATION x SESSION interaction χ(2) = 0.89, * p* = 0.640, f^2^ = 0.005
Sham	1.24 (0.13)	1.47 (0.12)	1.47 (0.16)	
**Required % of MSO to reach ~ 1 mV adjusted MEPs (% of MSO)**				
Active	56.07 (4.27)	56.60 (3.94)	58.40 (4.09)	STIMULATION χ(1) = 0.94, * p* = 0.332, f^2^ = 0.001; SESSION χ(2) = 2.63, * p* = 0.268, f^2^ = 0.002; STIMULATION x SESSION interaction χ(2) = 0.39, * p* = 0.823, f^2^ < 0.001
Sham	55.80 (3.71)	56.33 (3.61)	56.93 (3.66)

### Short intracortical inhibition during movement preparation (SICI_move_)

**Figure 3:**
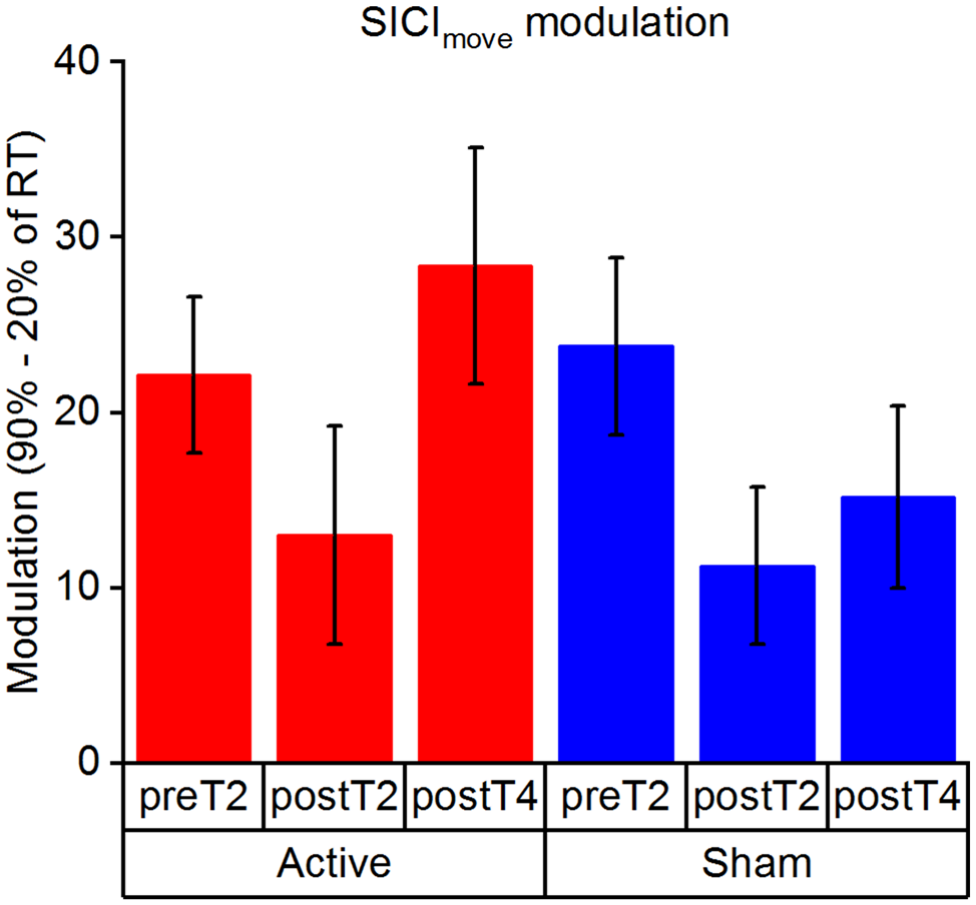
Modulation of SICI_move_, quantified by computing the delta between 90 and 20% of RT, during the time course of the protocol. Error bars are depicted as standard error of the mean (s.e.m.).

The analysis of the event-related SICI_move_ assessment indicated a significant effect of TIMING χ(1) = 48.08, *p* < 0.001, f^2^ = 0.174, confirming prior research^24,25^, which suggests disinhibitory dynamics of SICI towards movement onset. Subsequently, we analysed the modulation dynamics of SICI_move_. These were quantified by computing the difference of SICI_move_ assessed in late (90% of reaction time (RT)) and early premovement phase (20% of RT). We found a trend for effect of SESSION χ(2) = 5.37, *p* = 0.068, f^2^ = 0.013. There was not a significant effect of STIMULATION χ(1) = 1.23, *p* = 0.267, f^2^ = 0.057 or SESSION x STIMULATION interaction χ(2) = 2.37, *p* = 0.305, f^2^ = 0.024, see Fig. 3.

### SICI_move_ modulation and training performance

**Figure 4:**
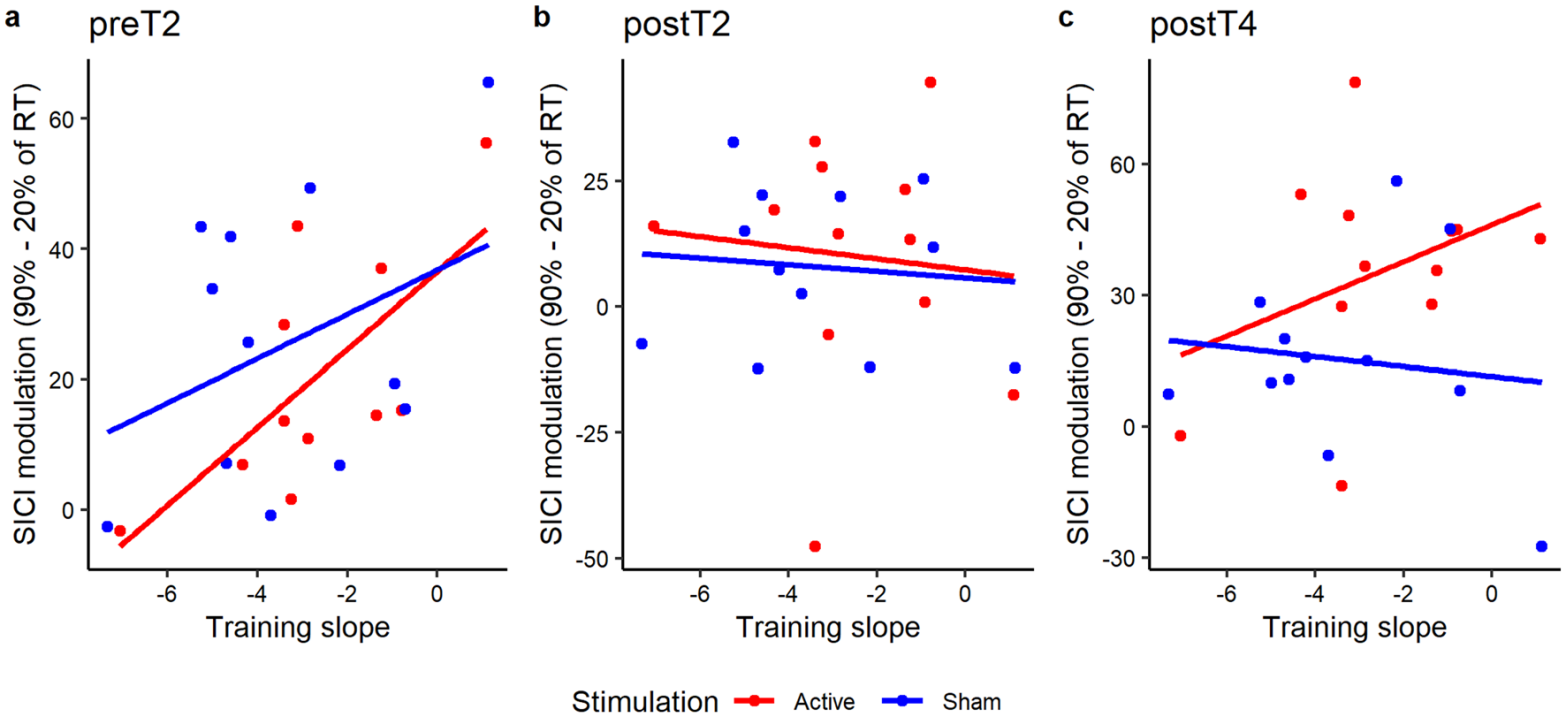
Association between SICI_move_ modulation and online learning, quantified by the linear slope fitted though the AUC data of the training blocks, for the protocol time points—(**a**) pre training (preT2), (**b**) post training (postT2), (**c**) post follow-up 2 (postT4). Lower values for training slope depict better learning. SICImove modulation is quantified by computing the delta between 90 and 20% of RT.

To further investigate underlying mechanisms, we assessed potential associations between SICI_move_ modulation and training success. For preT2 the results showed that there was a significant effect of MODULATION χ(1) = 5.73, *p* = 0.017, f^2^ = 0.296 showing that less modulation was associated with larger training success. There was no effect of STIMULATION χ(1) = 1.38, *p* = 0.241, f^2^ = 0.064 or MODULATION x STIMULATION interaction χ(1) = 0.87, *p* = 0.351, f^2^ = 0.040. During postT2 there was no significant MODULATION χ(1) = 0.15, *p* = 0.699, f^2^ = 0.007, STIMULATION χ(1) = 0.89, *p* = 0.345, f^2^ = 0.040 or MODULATION x STIMULATION interaction χ(1) = 0.02, *p* = 0.897, f^2^ < 0.001. At postT4 there was no significant effect of MODULATION χ(1) = 0.82, *p* = 0.364, f^2^ = 0.036, STIMULATION χ(1) = 0.36, *p* = 0.547, f^2^ = 0.016 or MODULATION x STIMULATION interaction χ(1) = 1.31, *p* = 0.252, f^2^ = 0.058. Please see Fig. 4.

### Rs-fMRI-based connectivity

**Figure 5:**
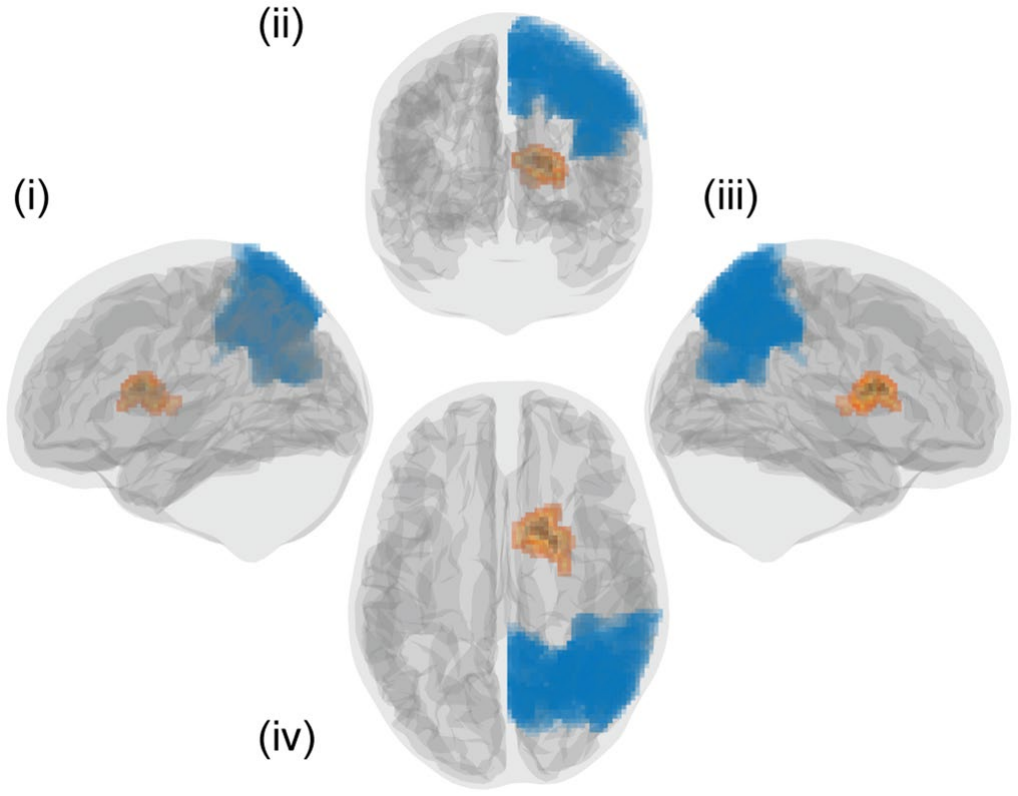
Effect on striato-parietal FC. Depicted is a cluster of voxels located in the right caudate (orange), which showed a significant TIME x STIMULATION interaction with a seed located in the parietal region (blue). (i) left, (ii) posterior, (iii) right, and (iv) superior view.

The analysis of seed-based FC revealed a significant TIME x STIMULATION interaction for a cluster of 226 voxels located in the right caudate with the seed in posterior parietal region (PPC), please see Fig. 5. Post-hoc analysis suggested a significant reduction of seed-based FC in the active group compared to the sham group in the 1^st^ follow-up session (T3), see Table 2. This reduction of seed-based FC in the active group in T3 was also significantly different from baseline (T1). Taking also into account the influential point analysis, our dataset failed to reveal clear associations between the modulation of seed-based FC between T1 and T3 and behavioural output (online learning, 24 h and 10d retention), please see Supplementary Table S2. For all other seeds, we did not find a significant TIME x STIMULATION interaction.

**Table 2:** Modulation of striato-parietal FC.

Comparison	T (df)	*p*-value, uncorrected	*p-*value, Bonferroni-corrected	Cohen’s *d*
Pre_active vs. pre_sham	1.28 (14)	0.222	0.888	0.33
Post_active vs. post_sham	− 6.97 (14)	< 0.001	< 0.001	1.80
Pre_active vs. post_active	6.22 (14)	< 0.001	< 0.001	1.61
Pre_sham vs. post_sham	− 2.16 (14)	0.048	0.192	0.56

Post-hoc testing for the modulation of seed-based FC for the significant cluster of voxels located in the right caudate and the PPC seed.

### Predicting training success

In order to study the predictive potential of behavioural, dpTMS, and rsfMRI measures for the dependent variable training success, we computed a linear mixed effects model. Training success was quantified by fitting a linear slope through the AUC data of the training phase. At first, we included the following fixed effects in the model: (i) BASELINE—performance in the SGFMT at the baseline block, (ii) MOTOR—compound motor score of the applied behavioural tests (pinch-, key-, grip force test, nine-hole peg test (9HPT), and the box and block test (BBT)) defined by the first two components extracted by principal component analysis, (iii) SICI_rest_ at preT2, (iv) ICF_rest_ at preT2, (v) ΔSICImove MODULATION—defined as the difference in SICI_move_ modulation between 90 and 20% of RT at preT2, and (vi) mean z-transformed correlation coefficient between the significant cluster in the right caudate and the PPC seed at T1. Participants were included as random effects to account for the repeated measures. The best model was selected based on the Akaike information criterion (AIC). The final model was able to explain 47.68% (R^2^) of the variance in training success. It included the factors BASELINE, SICI_rest_, ICF_rest_ and ΔSICI_move_ MODULATION. BASELINE (F(1,23) = 6.85, *p* = 0.015) had a significant influence on training performance, with every unit increase in baseline AUC the linear slope became more negative by − 0.03, indicating more learning. ΔSICImove MODULATION had a significant influence on training performance (F(1,23) = 7.90, *p* = 0.010), with every unit increase in modulation the linear slope became more positive by + 0.05, indicating less learning. See Table 3.

**Table 3:** Results of final predictive model for training success.

	Model statistics				Confidence interval (95%)		ANOVA statistics	
	Variance (SD)	Parameters estimates (SE)	T	*p-*value	Lower	Upper	F (df)	*p-*value
**Random effects**								
Participants	0.00 (0.00)							
Residuals	2.52 (1.59)							
**Fixed effects**								
Intercept		0.78 (1.98)	0.39	0.698	− 3.26	4.82		
BASELINE		− 0.03 (0.01)	− 2.62	0.015*	− 0.05	− 0.01	6.85 (1,23)	0.015*
SICI_rest_		− 0.04 (0.03)	− 1.52	0.143	− 0.09	0.01	2.31 (1,23)	0.143
ICF_rest_		0.00 (0.01)	0.10	0.921	− 0.02	0.02	0.01 (1,23)	0.921
ΔSICI_move_ MODULATION		0.05 (0.02)	2.81	0.010*	0.01	0.08	7.90 (1,23)	0.010*

Linear mixed effects model analysis was conducted to identify the most influential factors for predicting training success. The full model included following fixed factors: (i) BASELINE—SGFMT performance in the baseline block, (ii) MOTOR—compound motor score of the applied behavioural tests (pinch-, key-, gripforce test, 9HPT and BBT) defined by the first two components extracted by principal component analysis, (iii) SICI_rest_ at preT2, (iv) ICF_rest_ at preT2, (v) ΔSICI_move_ MODULATION—defined as the difference in SICImove modulation between 90 and 20% of RT at preT2, and (vi) mean z-transformed correlation coefficient between the significant cluster in the right caudate and the PPC seed at T1. The final model was selected based on the AIC criterion and identified ΔSICI_move_ MODULATION and BASELINE as the most influential factors.

* depicts *p* < 0.05.

## Discussion

The main finding of the study was that 50 Hz tACS applied to the ipsilateral cerebellum did not enhance the acquisition or retention of a novel motor skill based on sequential grip force control. This contrasts earlier research, which has reported beneficial effects of a similar stimulation protocol on different aspects of motor function, specifically the adaptation to frequency variations during finger tapping or to some aspects of the WFMT^6,15^. Several factors may explain our null results.

When implementing the present learning task, we formulated two core requirements. It should have considerable similarity to activities of daily living, such as grasping of objects, and should have a translational potential for future studies recruiting patients with motor constraints. The SGFMT fulfilled these criteria. However, the task is likely less specific to cerebellar resources as classical cerebellum-dependent tasks, such as motor adaptation or finger-tapping-based paradigms^26,27^. In fact, grip force execution and their modulation rely additionally to the sensorimotor cortex and the cerebellum on frontal, prefrontal, supplemental motor, cingulate motor, and parietal areas^28,29^. This potential neuronal non-specificity may have prevented us from detecting constricted beneficial effects.

It is possible that the chosen stimulation frequency was suboptimal to facilitate the underlying neuronal processing of the task. It is assumed that Purkinje cells (PC) are a core responsive element for tES^6,11^ and that they are crucially involved in motor learning^30,31^. Indeed, PCs show oscillatory dynamics in the gamma range, however 50 Hz oscillations might not be the most relevant component. For instance, the peak of the frequency distribution of PCs simple spikes during upper limb movements is rather located at higher frequencies ranges (circa 100 Hz)^32^. Furthermore, a considerable proportion of PCs shows an intrinsic trimodal firing pattern, which is characterized by an monotonical increase of the firing rate from the beginning of the monotonic towards the start of the burst period (circa 40 Hz to circa 100 Hz)^33^. In fact, a set of recent cerebellar tACS studies tested higher frequency ranges and provided evidence that 70 Hz cerebello-motorcortical tACS can induce beneficial effects, when studying an isometric force modulation task^34–36^. We found rather an indication for disturbing effects of the 50 Hz stimulation frequency, when assessing task retention (whole group level analysis). This combined evidence points towards the direction that 50 Hz cerebellar tACS protocols may be suboptimal for boosting performance in motor tasks relying on sequential modulation of grip forces. Future research should address potential effects of task specificity and different stimulation frequency ranges in greater detail.

**Figure 6:**
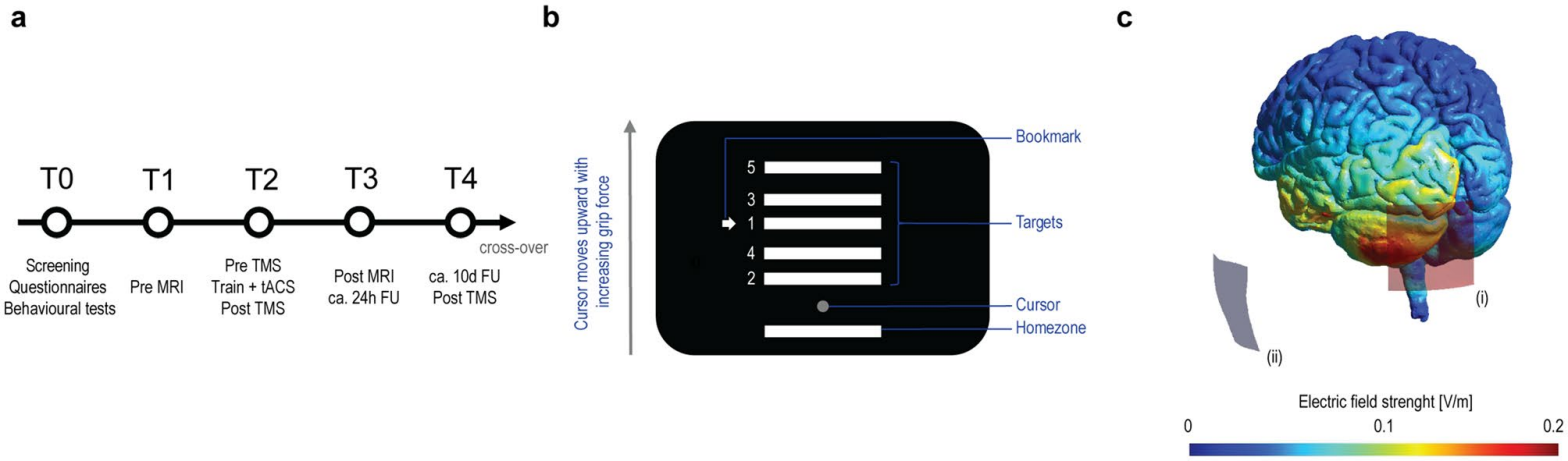
Experimental setup. (**a**) Timeline: tACS was applied in a double-blind, sham-controlled, cross-over design concurrently to the training (Train) of a novel motor skill (T2), skill retention was assessed at a circa 24 h and circa 10d follow-up (T3,T4), dpTMS and MRI assessments were incorporated in the experimental protocol (T1, T2, T3, T4). (**b**) Sequential grip force modulation task (SGFMT): participants had to navigate a cursor by modulating their applied grip force between a homezone and five target zones following a sequential order. (**c**) Computer simulation of the applied stimulation protocol implemented in SimNIBS^71^, the active electrode was placed 3 cm lateral to the inion over the left cerebellar hemisphere (i) and the return electrode over the ipsilateral buccinator muscle (ii)^11^, depicted is the electric field strength (norm E) at π/2 phase.

Furthermore, the stimulation site slightly differed from prior work from Naro and colleagues^6,15^—3 cm lateral to the inion over the left cerebellar hemisphere versus 1–2 cm below and 3–4 cm lateral to the inion over the right cerebellar hemisphere. When designing the study protocol, we modified the electrode montage according to the Celnik et al. configuration^11^ as it had shown efficiency in a similar motor task applying consecutive cerebellar tDCS^37^. Subsequently, our chosen montage may have influenced the magnitude of potential behavioural effects. However, when considering the focality range of conventional cerebellar tES protocols^38^, we consider this effect rather as negligible, see also Fig. 6C.

An additional point to consider is the site of the cerebellar hemisphere, which was stimulated (right versus left). We chose to train the left hand and stimulate the left cerebellar hemisphere to allow more room for improvement in right-hand dominant participants. It is important to note, that the majority of tES protocols targeted the right cerebellar hemisphere so far—e.g., the majority of studies identified in the recent cerebellar tDCS meta-analysis from Oldrati and colleagues^39^ stimulated the right cerebellar hemisphere. However, some authors have suggested that motor learning functions are lateralized towards the left cerebellar hemisphere^40,41^, which was targeted in our study. Certainly, more research is needed to identify potential hemispheric differences for responsiveness towards cerebellar neuromodulation protocols.

Furthermore, we studied potential underlying mechanisms mediated by GABAergic and glutamatergic motor-cortical circuits by assessing SICI_rest_ and ICF_rest_. The effect of motor training protocols on SICI_rest_ and ICF_rest_ is discussed controversially. For SICI_rest_, a reduction^42,43^ or no changes^44,45^ after motor training have been reported. Influential factors might be the nature of the applied training task, the utilized hand (dominant vs. non-dominant)^43^, or the phase of learning^42^. We were not able to detect any learning or stimulation associated effects on SICI_rest_. This absence of effects for SICI_rest_ (GABA_A_ surrogate^17^) extends prior work from Naro and colleagues, who found no effects of the applied protocol on the long-interval intracortical inhibition (GABA_B_ surrogate)^6,17^. For ICF_rest,_ the available data on motor training-related effects is more sparse, mainly no clear effects have been reported^46–48^. This is in agreement with our study, in which ICF_rest_ was not significantly modulated by training or stimulation.

We were able to show disinhibitory dynamics of SICI_move_ towards movement onset confirming prior research^24,25,49^. Our analysis suggested that these disinhibitory dynamics might be modulated by the phase of training, pointing towards a reduced modulation of SICI_move_ in the immediate post training phase. Contrary to our finding, Dupont-Hadwen and colleagues did not detect learning phase-dependent effects on SICI_move,_ when studying a ballistic thumb abduction task^49^. The differential results might be explained by task-dependent (simple reaction time vs. ballistic thumb abduction), or timing-dependent effects (timing of late premovement assessment 90% vs. 75%), or the nature of the provided feedback. However, in line with Dupont-Hadwen we were able to associate the magnitude of event-related disinhibition with the amount of learning, linking weaker SICI_move_ modulation with greater training-related improvement. We speculate that less disinhibitory dynamics in premovement phase are a correlate of a well-tuned motor system with effective inhibitory control and high susceptibility towards training success. Conversely, Heise and colleagues were able to link stronger event-related modulation of SICI_move_ with better motor performance in a life span cohort^25^, which however might be interpreted as a correlate of a compensatory mechanism to diminish age-related constraints^24,25,50^.

To study potential effects on the underling brain networks, we assessed seed-based FC with rsfMRI. Modulations of resting state networks in the post-training phase have been previously characterized. For instance, Sami and colleagues have reported an engagement of the sensory-motor network in the late post-training phase (> 6 h) studying the serial reaction time task^51^. In our sample, the combination 50 Hz cerebellar tACS and motor training reduced FC between a cluster of voxels, located in the right caudate, and the PPC seed. Functionally, preserved synchrony between cortical and striatal areas including parietal regions, has been associated with sleep-dependent memory consolidation^52^. Based on these considerations, one is tempted to speculate that the limited retention (whole group analysis) in the active stimulation group might have been a correlate of perturbed striato-parietal connectivity. However, in the present data, no clear association between the magnitude of reduced FC and task retention was found.

Baseline performance in the SGFMT and SICI_move_ modulation before training appeared as most influential determining factors for subsequent training success. Specifically, these results indicated that worse baseline performance was associated to better training performance. This could be either explained by the phenomenon that higher motor variability can result in better motor performance due to an increase in exploratory behaviour^53^. Conversely, it could point towards possible ceiling effects, as the initially better performers might be limited for further improvement, due to constraints of the task. The second influential factor was the modulation of SICI_move_. The results showed that less SICI_move_ modulation before training was associated with larger training success. This result is in contrary to prior research, which has linked stronger event-related modulation of inhibition with better motor performance^25^. This discrepancy might be explained by task- or cohort-specific effects and should be further addressed in future work. As discussed above, we currently speculate that weaker modulation of inhibitory dynamics is a correlate of an efficient and well-tuned motor system and by this means a favourable prerequisite for efficient motor learning.

A few limitations of the present work have to be mentioned. The studied sample size is rather small. However, the participant number is in the same range as similar prior proof-of-principle work in the field^4, 13^. Moreover, our chosen cross-over design provides the advantage of higher statistical power as e.g., the referenced work. We would like to emphasize that the SGMFT is based on modulation of grip forces in a constrained range and the results should not be overgeneralized to other motor tasks. It is of note, that other motor learning entities, such as motor adaptation paradigms, have shown responsiveness towards different non-invasive cerebellar stimulation paradigms. For instance, Koch and colleagues could show that cerebellar theta-burst stimulation (TBS) can enhance the learning in a visuomotor adaptation task in healthy participants^54^. Importantly, the aforementioned cerebellar TBS protocol has shown to improve gait and balance functions in chronic stroke patients^55^ pointing towards a considerable potential for successful further clinical translation. Lastly, it is important to note that fMRI-based FC studies have a poor test–retest reliability^56^. The described timing-dependent modulation of striato-parietal FC should be interpreted with caution.

Overall, cerebellar 50 Hz tACS did not enhance the acquisition or retention of a novel motor skill. Minor effects on striato-parietal FC were present. By means of linear mixed effects modelling, we were able to explain circa 48% of the variance in training success, and identified baseline task performance and the modulatory dynamics of SICI_move_ as the most influential determining factors. Accounting for the identified most influential factors may allow to stratify participants for future training-based interventional studies.

## Methods

### Participants

We recruited N = 15 young, healthy, right-handed participants for the study applying a cross-over design (N = 7 female, mean age ± s.d.: 26.20 ± 3.34, mean laterality quotient Edinburgh handedness inventory 89.47^57^). 24 data sets were considered for the analysis including behavioural measures, the remaining data sets were excluded due to application of a preliminary version of the motor learning task (N = 5) and technical difficulties during recording (N = 1). Our inclusion criteria were: ≥ 18 and < 35 years, right-handedness, normal values of Mini-mental state examination (> 26/30), absence of contraindication for transcranial electric stimulation (tES), transcranial magnetic stimulation (TMS), or magnetic resonance imaging (MRI). Our exclusion criteria were: presence of neuropsychiatric diseases, history of seizures, intake medication that potentially interacts with tES or TMS, musculoskeletal dysfunction that compromise finger movement, pregnancy, professional musician or intense professional usage of a computer keyboard, intake of narcotic drugs, request of not being informed in case of incidental findings. The study was carried out in accordance to the Declaration of Helsinki^58^. Written informed consent was obtained from all participants. Approval was obtained from the cantonal ethics committee Vaud, Switzerland (project number 2017–00765).

### Experimental design

The study followed a double-blind, sham-controlled, cross-over design. At session T0 participants were screened, filled in baseline questionnaires, and were characterized with a set of behavioural tests, including pinch-, key-, grip-force assessments, the nine-hole peg test (9HPT), and the box and block test (BBT). At session T1, we acquired a baseline MRI scan. Session T2 consisted of the motor training with concurrent tACS. The training session was embedded in dpTMS assessments (preT2 and postT2). Task retention was assessed 1 day and circa 10 days after the training phase (T3 and T4). Additionally, at T3 a follow-up MRI assessment (preT3) and at T4 a follow-up dpTMS assessment (postT4) were conducted. The minimum time between T4 before cross-over and T1 after cross-over was set to two weeks. See also Fig. 6a.

### Motor learning task

As a motor skill learning task, we used a computerized sequential grip force modulation task (SGFMT) adapted from Reis and colleagues^4^, implemented in MATLAB (The MathWorks, Inc., Natick, MA, USA), see Fig. 6b. The grip forces were sampled with a fibre optic grip force sensor (Current designs, Inc., Philadelphia, PA, USA). The participants had to control an onscreen cursor via the modulation of grip forces using their non-dominant, left hand. The cursor moved upwards in vertical direction with increasing forces. The participants were instructed to navigate the cursor between a homezone (H) and 5 target zones, which were scaled to individual maximum force. A rightward pointing arrow (bookmark) indicated which target had to be reached next. The instruction was to perform the task as accurately and as quickly as possible. In the training session (T2), the participants were firstly familiarized to the task (simplified task version, only 3 target zones). Following the familiarization, they conducted a 90 s baseline without tACS. The actual training with concurrent tACS lasted circa 20 min and consisted of 9 blocks. The follow-up sessions consisted of 3 blocks. The blocks lasted 90 s and were separated by breaks. The targets followed the sequential order A or B counterbalanced between the active and sham stimulation condition, see also Supplementary Table S3. However, in Block 5 the targets were arranged in a pseudorandom, untrained order, to assess for effects on simple motor performance.

### Cerebellar tACS

tACS was applied to the left cerebellum utilizing a DC-stimulator plus (neuroConn GmbH, Ilmenau, Germany). The stimulation protocol was adapted from Naro and colleagues^6^ and was defined by following parameters: sinusoidal waveform, intensity 2 mA (peak-to-peak), fade-in/out interval 2 s (100 × 2π cycles), duration (i) active 20 min (60,000 × 2π cycles) (ii) sham 30 s (cycles: 1,500 × 2π), 5 × 5 cm rectangular sponge-covered conductive rubber electrodes soaked in saline solution. The active electrode was placed 3 cm lateral to the inion and the return electrode over the ipsilateral buccinator muscle^11^, please see Fig. 6C.

### Double-pulse transcranial magnetic stimulation (dpTMS)

DpTMS was used to study GABAergic and glutamatergic circuits in the motor cortex^17^ at baseline and their modulation after stimulation and during the time course of learning. We assessed short intracortical inhibition at rest (SICI_rest_), during movement preparation (SICI_move_) and intracortical facilitation at rest (ICF_rest_)^24,59^. Methods are described in detail in our prior published work^24,60^. In brief, monophasic TMS pulses inducing a posterior to anterior current direction in the underlying brain tissue were applied using a MagPro X100 stimulator connected to a MC-B70 coil (MagVenture, Farum, Denmark). The coil was placed over the hotspot of the first dorsal interosseous muscle (FDI) with its handle pointing backwards circa 45° to the midsagittal line. The coil positioning was guided throughout the experiment with the support of a neuronavigation system (Localite, Bonn, Germany). The conditioning pulses (CP) were adjusted to 80% of resting-motor threshold (RMT), defined as the lowest stimulus intensity that produced a motor-evoked potential (MEP) with a peak-to-peak amplitude ≥ 50 μV in 5 out of 10 consecutive trials^61^. The test pulse (TP) was adjusted to elicit an MEP of circa 1 mV in the relaxed FDI. Stimulator output intensities for TP and CP were readjusted before each session to measure SICI/ICF magnitude modulation in a comparable part of the respective recruitment curves^62,63^, with the aim to control for the confound of potential changes in cortico-spinal excitability. SICI and ICF were assessed with the following parameters and tested together with test pulse only trials following a pseudorandom order: inter-stimulus interval (i) SICI 3 ms (ii) ICF 10 ms, number of trails per condition (i) rest 18 (ii) SICI_move_ 24, inter-trail-interval (i) rest 7 s ± 25% (ii) SICI_move_ 5.5, 6.5, 7.5, 8.5 s. During the SICI_move_ assessments, the participants performed a simple reaction time task (RT)^24^, which was implemented in Presentation software (Neurobehavioral Systems, Inc., Berkeley, CA, USA). The participants had to perform left index finger abductions as fast as possible after visual cue (circle). During the inter-trial-interval they we instructed to fixate a fixation cross with their eyes. The TMS pulses were applied at 20 and 90% of individual median RT. The electromyography (EMG) data as sampled with a Noraxon DTS Receiver (Scottsdale, AZ, United States) with the following settings: gain 500, sampling rate 3,000 Hz, high-pass filter 10 Hz analog Sallen-Key, low-pass filter 1,000 Hz digital FIR 128th order Butterworth. The signal was transferred for further processing and saved on a laptop via Signal software (Cambridge Electronic Design, Ltd., Cambridge, UK).

### Magnetic resonance imaging (MRI)

The MRI data were obtained with a Prisma 3 T scanner (Siemens Healthcare AG, Erlangen, Germany). Structural T1-weighted images were obtained with following parameters: 208 slices, voxel size 1 × 1 × 1 mm, 8° flip angle, repetition time (TR) 2,300 ms, echo time (TE) 2.26 ms, 256 mm field of view. Whole brain echo-planar images (EPI) were acquired at resting-state with following acquisition parameters: duration 7:11 min, 66 slices, 420 volumes, voxel size 2 × 2 × 2 mm, 50° flip angle, TR 1,000 ms, TE 32 ms, 225 mm field of view. During the acquisition of the EPI the participants were instructed to keep their eyes open and fixate a fixation cross.

### Data processing

The behavioural motor learning data were processed with an in-house script implemented in MATLAB. Our a priori primary outcome was defined as the area under the curve (AUC) of the movement trajectory for correctly performed sequences. For further processing the motor learning data was averaged per block.

The MEP data were visually inspected and processed by using an in-house MATLAB-based graphical user interface, which automatically documented trial rejections and processing steps, for trial rejection criteria please see Supplementary Information. The peak-to-peak MEP amplitude was measured in a response window of TMS pulse + 20 ms to + 50 ms and was averaged per condition. DpTMS conditions were contrasted to the corresponding TS only condition and expressed as mean (conditioned MEPs) / mean (unconditioned MEPs) ∗ 100.

The pre-processing of the rsfMRI data and the 1st level analysis was done using CONN toolbox^64^. We calculated seed-based FC by using each area in a hypothesis driven motor learning network^8^ as a seed, for further details see Supplementary Table S4.

### Statistical analysis

Statistical significance was assumed at *p*-values < 0.05. Normality of the data were confirmed by assessing their skewness, which range was in-between 1 and − 1^65^. The statistical analysis of the behavioural data, TMS data, and for possible biomarkers of training success was implemented in RStudio (version 1.1.456, 2018). Linear Mixed-Effects Models were fitted using the lme4 package^66^. Statistical testing was done with use of the likelihood ratio test^67^. To further examine potential null results of main behavioural outcomes as suggested by the frequentist analysis, we added in addition a Bayesian approach by computing Bayesian ANOVAs in JASP software (Version 0.9.1, 2018)^68^. For the predictive model the goodness of fit was determined by the Akaike information criterion (AIC). The statistical analysis of the seed-based FC data was done by calculating RM-ANOVAs and was implemented in SPM12^69^, statistical significance was determined at a cluster-level threshold of FDR-corrected *p-*value < 0.05 with a voxel-level threshold of uncorrected *p*-value < 0.001. Post-hoc analysis for the modulation of functional connectivity was done with paired t-tests, Bonferroni-corrected. Effect sizes for the linear mixed-effect models were expressed as Cohen’s f^2^ (≥ 0.02 small, ≥ 0.15 medium, ≥ 0.35 large) based on the approach of Selya and colleagues^70^ and as Cohen’s *d* (≥ 0.2 small, ≥ 0.5 medium, ≥ 0.8 large) for the t-tests. The values in figures are shown as mean ± standard error of the mean (s.e.m.).

## Supplementary information

Supplementary file1

## Data Availability

The datasets generated during and/or analysed during the current study are available from the corresponding author on reasonable request.
