# Supplementary material for: Cerebellar transcranial alternating current stimulation in the gamma range applied during the acquisition of a novel motor skill
Source: Sci Rep. 2020 Jul 8;10:11217. doi: 10.1038/s41598-020-68028-9 (PMC7343806; doi:10.1038/s41598-020-68028-9)
Supplement: Supplementary file 1 — Supplementary file1 [file 41598_2020_68028_MOESM1_ESM.pdf]

# Supplementary Online Material: Cerebellar transcranial alternating current stimulation in the gamma range applied during the acquisition of a novel motor skill

Maximilian J. Wessel[1,2#*], Laurijn R. Draaisma[1,2#], Anne F. W. de Boer[1,2], Chang-Hyun Park[1,2], Pablo Maceira-Elvira[1,2], Manon Durand-Ruel[1,2], Philipp J. Koch[1,2], Takuya Morishita[1,2] & Friedhelm C. Hummel[1,2,3]

[#]authors contributed equally

[1] Defitech Chair of Clinical Neuroengineering,
Center for Neuroprosthetics (CNP) and Brain Mind Institute (BMI),
Swiss Federal Institute of Technology (EPFL), Geneva, Switzerland
[2] Defitech Chair of Clinical Neuroengineering,
Clinique Romande de Réadaptation,
Center for Neuroprosthetics (CNP) and Brain Mind Institute (BMI),
Swiss Federal Institute of Technology (EPFL Valais), Sion, Switzerland
[3] Clinical Neuroscience, University of Geneva Medical School, Geneva, Switzerland

*Correspondence:
Dr. Maximilian J. Wessel
Defitech Chair of Clinical Neuroengineering
Center for Neuroprosthetics (CNP) and Brain Mind Institute (BMI)
Swiss Federal Institute of Technology (EPFL)
Campus Biotech,
Chemin des Mines 9
1202 Geneva
email: maximilian.wessel@epfl.ch
Tel: +41 21 695 50 90

## Transcranial alternating current stimulation (tACS)-associated sensations

**Active versus sham stimulation distinction.** Participants could not effectively distinguish the active from the sham stimulation during the two sessions. The proportion of correct distinctions before cross-over was .467 and was not significantly different from chance level $p$ = 1.00. The proportion of correct distinctions after cross-over was .600 and not significantly different from chance level $p$ = 0.607. The respective statistical analysis was implement in JASP[1].

**Reported sensations.** After the final training session, the participants were asked about their perceived sensations during the stimulation for both training sessions applying a structured questionnaire, adapted from Antal and colleagues[2]. We checked for the following sensations: itching, pain, burning, metallic/iron taste in mouth, warmth, fatigue, other. Response options were: “0” = none, “1” = mild, “2” = moderate, “3” = strong. Most of the responses for all of the sensations were either “none” or “mild”. None of the reported sensations differed significantly between active and sham stimulation, please see also table below. The respective statistical analysis was implement in JASP[1].

| | **None** | | **Mild** | | **Moderate** | | **Strong** | | **Statistics** | |
|---|---|---|---|---|---|---|---|---|---|---|
| | **Active** | **Sham** | **Active** | **Sham** | **Active** | **Sham** | **Active** | **Sham** | **Chi-square** | ***p*-value** |
| Itching | 33.3 | 33.3 | 53.3 | 60 | 13.3 | 6.7 | 0 | 0 | 0.39 | .822 |
| Pain | 80 | 73.3 | 20 | 26.7 | 0 | 0 | 0 | 0 | 0.19 | .666 |
| Burning | 53.3 | 66.7 | 40 | 20 | 0 | 13.3 | 6.7 | 0 | 4.22 | .238 |
| Warmth | 60 | 40 | 33.3 | 60 | 6.7 | 0 | 0 | 0 | 2.74 | .254 |
| Metallic/iron taste | 100 | 100 | 0 | 0 | 0 | 0 | 0 | 0 | n/a | n/a |
| Fatigue | 80 | 86.7 | 6.7 | 13.3 | 13.3 | 0 | 0 | 0 | 2.37 | .305 |
| Other | 40 | 53.3 | 26.7 | 26.7 | 33.3 | 20 | 0 | 0 | 0.79 | .675 |

Numbers correspond to percentage of participants, who chose the response option for the respective stimulation condition.

**Supplementary Table S1.** tACS-associated sensations depicted for the active and sham stimulation condition separately.

## No clear association between modulation of striato-parietal FC and behaviour

| | **SBC** | | | **SBC x STIMULATION Interaction** | | |
|---|---|---|---|---|---|---|
| | **χ(df)** | ***p*-value** | **$f^2$** | **χ(df)** | ***p*-value** | **$f^2$** |
| Online learning | *3.60(1)* | *p* = .058 | $f^2$ = *.169* | *0.94(2)* | *p* = .624 | $f^2$ = *.017* |
| Retention 24h | *0.24(1)* | *p* = .621 | $f^2$ = *.009* | *7.27(2)* | *p* = .026 | $f^2$ = *.094* |
| Retention 24h (influential point analysis) | *0.35(1)* | *p* = .552 | $f^2$ = *.016* | *1.66(2)* | *p* = .437 | $f^2$ = *.069* |
| Retention 10d | *0.01 (1)* | *p* = .913 | $f^2$ = < *.001* | *6.54(2)* | *p* = .038 | $f^2$ = *.028* |
| Retention 10d (influential point analysis) | *0.01(1)* | *p* = .904 | $f^2$ = < *.001* | *2.60(2)* | *p* = .273 | $f^2$ = *.018* |

**Supplementary Table S2.** Three separate linear mixed-effect models were calculated for online learning, 24h retention and 10d retention to assess whether the change in SBC and SBC x STIMULATION interaction had a significant influence. The behavioural data was taken as the dependent variable and the delta between SBC T1 and T3 was used, as well as the stimulation effect. In line with the analysis reported in the main manuscript, an influential point analysis was conducted for the retention variables.

## Applied sequences in the sequential grip force modulation task (SGFMT)

| **Block** | **Sequence** |
|---|---|
| Baseline A | H-4-H-3-H-1-H-2-H-5 |
| Baseline B | H-5-H-4-H-2-H-3-H-1 |
| Training A | H-3-H-1-H-4-H-2-H-5 |
| Training B | H-2-H-1-H-5-H-3-H-4 |
| Pseudorandom A | H-1-H-5-H-2-H-4-H-3 |
| Pseudorandom B | H-4-H-2-H-5-H-1-H-3 |
| H: homezone, numbers correspond to bar position on the screen counting from the home zone upwards. | |

**Supplementary Table S3.** The order of targets followed predefined, complexity-matched sequences. Sequence set A or B was allocated to one of the two stimulation conditions following a pseudorandom order.

## TMS trial rejection criteria

Trial rejection criteria were as follows: muscle preactivation exceeding ± 25 µV from baseline < 100 ms in resting-state or ± 50 µV for event-related trials before and in an 20 ms interval following the TMS pulse, muscle preactivation ± 100 µV from baseline from start of the trial to 100 ms before the TMS pulse, no clear MEPs in the $TP_{only}$ and ICF conditions (defined as peak-to-peak amplitude < 50 µV), overlap of the MEP with the voluntary muscle activation in event-related trials, or trials with documented suboptimal coil placement.

## Applied parcellations for defining the regions of interests (ROIs) of the hypothesis-driven motor learning network and the corresponding seeds

| ROI | Side | Atlas | Name of ROI according to atlas | Reference |
|---|---|---|---|---|
| Primary motor cortex (M1) | Right | Brainnetome atlas | Upper limb | [4] |
| Dorsal premotor cortex (PMd) | Right | Brainnetome atlas | PMd | [4] |
| Ventral premotor cortex (PMv) | Right | Brainnetome atlas | PMv | [4] |
| Supplementary motor area (SMA) | Right | AAL | SMA | [5] |
| Primary somatosensory cortex (S1,2,3) | Right | Brodmann atlas | Area 1, 2 and 3 | [6] |
| Dorsolateral prefrontal cortex (DLPFC) | Right | Brainnetome atlas | A9 and 46 (dorsal area of the middle frontal gyrus) | [4] |
| Motor area of the thalamus (THAL) | Right | Behrens atlas | Motor part | [7] |
| Posterior parietal cortex (PPC) | Right | Brodmann atlas | Area 5, 7, 39 and 40. Contains inferior and superior parietal cortex | [6] |
| Cerebellum (CB) | Left | Bruckner atlas | Network 2 | [8] |
| Striatum (STRIAT) | Right | Choi atlas | Network 2 | [9] |

**Supplementary Table S4.** Definition of hypothesis-driven ROIs of the assessed motor learning network.

## References


1. JASP Team. *JASP (Version 0.8.5.1).* (2019). URL https://jasp-stats.org/.
2. Antal, A. *et al.* Low intensity transcranial electric stimulation: Safety, ethical, legal regulatory and application guidelines. *Clin Neurophysiol* **128**, 1774–1809 (2017).
3. Hummel, F. C. *et al.* Deficient intracortical inhibition (SICI) during movement preparation after chronic stroke. *Neurology* **72**, 1766–72 (2009).
4. Fan, L. *et al.* The Human Brainnetome Atlas: A New Brain Atlas Based on Connectional Architecture. *Cereb. Cortex* **26**, 3508–3526 (2016).
5. Tzourio-Mazoyer, N. *et al.* Automated anatomical labeling of activations in SPM using a macroscopic anatomical parcellation of the MNI MRI single-subject brain. *Neuroimage* **15**, 273–89 (2002).
6. Rorden, C. & Brett, M. Stereotaxic display of brain lesions. *Behav Neurol* **12**, 191–200 (2000).
7. Behrens, T. E. *et al.* Non-invasive mapping of connections between human thalamus and cortex using diffusion imaging. *Nat Neurosci* **6**, 750–7 (2003).
8. Buckner, R. L., Krienen, F. M., Castellanos, A., Diaz, J. C. & Yeo, B. T. T. The organization of the human cerebellum estimated by intrinsic functional connectivity. *J. Neurophysiol.* **106**, 2322–2345 (2011).
9. Choi, E. Y., Yeo, B. T. T. & Buckner, R. L. The organization of the human striatum estimated by intrinsic functional connectivity. *J. Neurophysiol.* **108**, 2242–2263 (2012).
